# Learning curve for robotic-assisted total knee arthroplasty

**DOI:** 10.1186/s13018-026-06835-y

**Published:** 2026-04-06

**Authors:** Filippo Migliorini, Luise Schäfer, Ludovico Lucenti, Jens Schneider, Andrea Maria Nobili, Daniel Kämmer, Nicola Maffulli, Andreas Bell

**Affiliations:** 1 Department of Trauma and Reconstructive Surgery, University Hospital of Halle, Martin-Luther University Halle-Wittenberg, Ernst-Grube-Street 40, 06097 Halle (Saale), Germany; 2 Department of Orthopaedic and Trauma Surgery, Eifelklinik St. Brigida, Kammerbruschstr. 8, 52152 Simmerath, Germany; 3 Department of Orthopaedic and Trauma Surgery, Academic Hospital of Bolzano (SABES-ASDAA), via Lorenz Böhler 5, Bolzano, 39100 Italy; 4 Department of Life Sciences, Health, and Health Professions, Link Campus University, Via del Casale di San Pio V, Rome, 00165 Italy; 5 Department of Precision Medicine in MedicalSurgical and Critical Care (Me.Pre.C.C.), University of Palermo, Palermo, 90133 Italy; 6 Faculty of Medicine and Psychology, University “La Sapienza” of Rome, Rome, Italy; 7 School of Pharmacy and Bioengineering, Keele University Faculty of Medicine, ST4 7QB Stoke on Trent, UK; 8 Centre for Sports and Exercise MedicineBarts and the London School of Medicine and Dentistry, Queen Mary University of London, Mile End Hospital, 275 Bancroft Road, London, E1 4DG UK

**Keywords:** Navigation, Replacement, Biomechanics, Surgery, Osteoarthritis, Surgical time, Computational

## Abstract

**Introduction:**

The learning curve refers to the relationship between a learner's execution of a task and the number of attempts or time necessary to perform it in a predictable, reliable, and optimal fashion. The learner's competence in a task should improve over time as they execute the job more frequently. The present investigation aims to clarify the learning curve associated with robotic-assisted total knee arthroplasty (TKA).

**Methods:**

Consecutive patients undergoing total knee arthroplasty at the Department of Orthopaedic Surgery, Eifelklinik St. Brigida, Simmerath, Germany, between 2021 and 2025 were prospectively screened for participation in this clinical study. All procedures were performed through a medial parapatellar approach, following a functional alignment strategy. Implantation was performed in accordance with the manufacturer’s recommendations using the Smith & Nephew Legion Genesis II system with a posterior-stabilised polyethylene insert. Both femoral and tibial components were cemented with Palacos cement (Heraeus Medical GmbH, Wehrheim, Germany). Postoperative physiotherapy followed the standard institutional protocol. At hospital admission, demographic variables including age, body mass index (BMI), and sex were recorded. Operative time was documented for each procedure and defined as the interval from skin incision to completion of wound closure.

**Results:**

The first 200 robotic-assisted TKAs were monitored. 66% (112 of 200 patients) were women, and 47.5% (95 of 200 TKAs) were performed on the right side. The mean age of the patients was 68.6 ± 8.1 years, and their BMI was 28.6 kg/m². The exponential decay model revealed a characteristic learning curve, characterised by initial rapid gains followed by a plateau. The estimated asymptotic operative time was approximately 89.2 minutes, with a learning rate coefficient of 0.035. This implies that the majority of efficiency improvements occur early, but meaningful reductions persist beyond the 20th case. Block-wise comparisons supported the existence of an earlier functional learning threshold. Statistically significant reductions in operative time, compared with the first 10 cases, were observed from the 41st to 50th procedure block (p = 0.02), with stabilisation in the 90-minute range thereafter.

**Conclusion:**

The most efficient gains occur early, and operative times stabilise at around 90 minutes after approximately 40 procedures.

***Registration*:**

German Registry of Clinical Trials (ID DRKS00030614).

## Introduction

Symptomatic knee osteoarthritis (OA) is common and adversely impacts both the quality of life and participation in activities for affected patients [1, 2]. In patients with end-stage knee osteoarthritis, total knee arthroplasty (TKA) may become an essential therapeutic intervention [3, 4]. Despite advances in implant design and surgical technique, TKA continues to have a low rate of unsatisfactory outcomes, even when components are perfectly implanted and alignment is achieved [5–7]. Many patients still report dissatisfaction after TKA, and in some cases, the cause of failure or persistent symptoms remains unexplained, highlighting the complexity of factors influencing patient outcomes beyond surgical precision [8, 9]. This ongoing challenge provides fertile ground for the adoption of robotic-assisted TKA, which aims to enhance accuracy, reduce variability, and potentially improve clinical outcomes by leveraging advanced technology for more individualised and precise component placement [10, 11]. However, despite many advantages, concerns remain regarding costs, increased operative time, the absence of long-term follow-up, and, predominantly, the learning curve associated with this technique [12, 13]. The learning curve refers to the relationship between a learner’s performance on a task and the number of attempts or time required to perform it [14]. The learner’s competence in a task should improve over time as they execute the job more frequently [15].

The CORI system (Smith & Nephew Ing, London, UK) is an imageless, handheld robotic platform designed to support intraoperative anatomical mapping and navigated bone preparation during TKA [16]. Unlike image-based systems, CORI does not require preoperative computer tomographic (CT) or magnetic resonance imaging (MRI) scans, relying instead on intraoperative data acquisition to produce a 3D model of the joint [16, 17]. The system facilitates dynamic assessment of soft-tissue balance throughout the range of motion, thereby supporting personalised alignment strategies and surgical decision-making [18]. The present investigation aims to characterise the learning curve associated with robotic-assisted TKA, with a specific focus on operative efficiency.

## Methods

### Study design and setting

The present study constitutes a predefined secondary analysis of a prospectively registered clinical cohort. Details of the original study protocol have been published previously [19]. The present investigation addresses a distinct research question focusing on the learning curve of robotic-assisted total knee arthroplasty.

### Study design

Consecutive patients undergoing primary total knee arthroplasty at the Department of Orthopaedic Surgery, Eifelklinik St. Brigida (Simmerath, Germany), between 2021 and 2025 were prospectively screened for inclusion in this study. The investigation was performed in accordance with the Declaration of Helsinki. The study protocol was registered in the German Clinical Trials Register (DRKS00030614) and approved by the Ethics Committee of the North Rhine Medical Council, Düsseldorf, Germany (ID 2022374).

### Eligibility criteria

Eligible patients were adults (≥ 18 years) with symptomatic knee OA graded II to IV according to the Kellgren–Lawrence classification [16], who were able to provide informed consent. Patients were excluded in the presence of relevant medical conditions or protocol deviations that could interfere with study outcomes, including inflammatory or neoplastic disease, pregnancy or breastfeeding, coagulation disorders, relevant neurological or vascular pathology, peripheral ulcerations, incomplete endpoint data, non-standardised postoperative blood sampling, inability to comply with the postoperative management protocol, or any other condition considered capable of influencing the results.

### Surgical technique

A standardised preoperative clinical, imaging, and anaesthesiological pathway was applied to all patients. Perioperative antibiotic prophylaxis consisted of a single intravenous administration of 1.5 g cefuroxime at induction of anaesthesia. Postoperative analgesia was provided by a continuous femoral nerve block maintained for 48 h. All procedures were performed by a single surgeon (AB) through a medial parapatellar approach following a functional alignment concept. Prosthetic components were implanted according to the manufacturer’s recommendations using the Smith & Nephew Legion Genesis II system with a posterior-stabilised polyethylene insert, and both femoral and tibial components were cemented with Palacos cement (Heraeus Medical GmbH, Wehrheim, Germany). One deep closed-suction drain and one superficial subcutaneous drain were left in situ for 48 h. Thromboprophylaxis consisted of subcutaneous enoxaparin sodium (40 mg once daily) administered for six weeks, starting 12 h after surgery.

### Rehabilitation protocol

Postoperative rehabilitation followed the standard institutional physiotherapy pathway [21]. Patients were supervised daily by trained physiotherapists starting on the first postoperative day. In the absence of complications, discharge was planned after a minimum hospital stay of five days. From postoperative day 2, patients underwent two daily sessions of continuous passive motion lasting 60 min each, during which progressive increases in knee range of motion were applied. Discharge criteria included attainment of at least 80° of knee flexion. Early mobilisation and progressive range-of-motion exercises were initiated postoperatively, including assisted ambulation and stair training according to functional recovery. After discharge, all patients continued with an individualised inpatient or outpatient rehabilitation programme for at least three weeks. Patients with relevant deviations from the planned surgical or rehabilitation protocol were excluded from the analysis.

### Outcomes of interest

Baseline demographic data, including age, sex distribution, and BMI, were recorded at hospital admission. Operative time was the primary outcome of interest and was documented for each procedure. Surgical duration was defined as the interval from skin incision to completion of wound closure.

### Statistical analysis

All statistical analyses were performed using IBM SPSS Statistics version 25. For descriptive statistics, the arithmetic mean and standard deviation (SD) were used. The learning curve was evaluated by collecting operative times from the first robotic TKA onward. Two complementary statistical approaches were applied to ensure a robust and multidimensional understanding of the learning progression. An exponential curve-fitting model was used to describe the overall reduction in operative time across successive cases, employing the exponential decay function. This approach reflects the typical diminishing-return profile seen in surgical skill acquisition, where time improvements become asymptotically smaller. A block-wise comparison was also performed. The dataset was divided into sequential blocks of 10 procedures. For each block, the mean operative time was compared to the first block (cases 1–10) using Welch’s t-test for unequal variances, with values of *P* < 0.05 considered statistically significant. This approach allowed the detection of statistically significant reductions in operative time at specific procedural intervals.

## Results

### Patient demographics

The first 200 robotic-assisted TKAs were monitored. 66% (112 of 200 patients) were women, and 47.5% (95 of 200 TKAs) were performed on the right side. The mean age was 68.6 ± 8.1 years, and the mean BMI was 28.6 kg/m^2^.

### Outcomes of interest

**Fig. 1:**
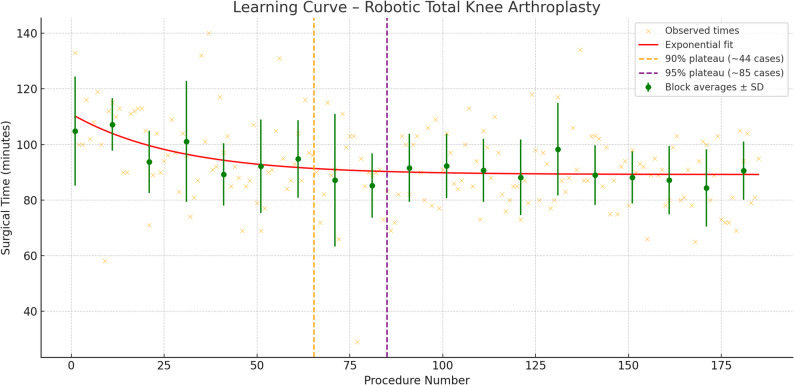
Exponential Model of Learning Curve. The curve shows a rapid decline in operative time in early cases, followed by a plateau at approximately 89 min

The exponential decay model revealed a characteristic learning curve, characterised by initial rapid gains followed by a plateau (Fig. 1). The estimated asymptotic operative time was approximately 89.2 min, with a learning rate coefficient of 0.035. This implies that the majority of efficiency improvements occur early, but meaningful reductions persist beyond the 20th case.

Block-wise comparisons supported the existence of an earlier functional learning threshold (Table 1). Statistically significant reductions in operative time, compared to the first 10 cases, were observed beginning from the 41st to 50th procedure block (*p* = 0.02), with stabilisation in the 90-minute range observed thereafter.

**Table 1:** Block-wise Statistical Comparison. P-values were calculated using Welch’s t-test comparing each block to the first 10 procedures

Block	Block 1	Block 2	Block 3	Block 4	Block 5	Block 6
2	104.80	107.20	-	-	-	-
3	104.80	-	93.70	-	-	-
4	104.80	-	-	101.10	-	-
5	104.80	-	-	-	89.20	-
6	104.80	-	-	-	-	92.20

## Discussion

According to the main findings of the present investigation, the most efficient gains occur early, and operative times stabilise at around 90 min after approximately 40 procedures.

The authors identified only one clinical study which specifically evaluated the learning curve of the CORI robotic-assisted system [15]. Weaver et al. [15] conducted a retrospective analysis of 500 robotic-assisted TKAs performed with CORI between October 2021 and February 2023, comparing them to 150 conventional freehand TKA procedures. Using Cumulative Sum (CUSUM) analysis, the authors identified a learning curve threshold of only six cases [15]. Operative times were initially longer for robotic procedures but reached equivalence with conventional techniques by the sixth case, indicating a rapid acquisition of procedural efficiency [15]. Furthermore, technical reports have described that the CORI system streamlines intraoperative workflow by automatically capturing anatomical landmarks, reducing the number of surgical steps by 40%, and decreasing bone resection time by approximately 29% [15]. These workflow enhancements may lead to a shorter, more manageable learning curve than with other robotic systems. The findings of the present investigation suggest a substantially longer learning curve, extending to approximately 40 procedures. Several factors may explain this discrepancy. First, differences in study design and methodology, particularly the definition and statistical modelling of “proficiency,” may influence the identified inflexion point. Second, variations in surgical technique, integration of institutional workflows, and perioperative team familiarity with robotic protocols can significantly affect the early operative experience. Third, the inclusion of more complex or diverse patient anatomies in our cohort may have introduced variability, thereby prolonging the adaptation phase. Finally, although the surgeon in our study had extensive prior experience in knee arthroplasty and robotic training, the specific dynamics and user interface of the CORI system may still require a more extended adjustment period before achieving procedural efficiency and consistency across different case types. The current evidence on the learning curve for other robotic systems is also heterogeneous [20]. In a retrospective analysis of 101 patients who underwent robotic-assisted TKA using the NAVIO system, Bosco et al. [21] reported a mean operative time of 72 ± 18.4 min. Using segmented regression analysis, they found that procedural accuracy remained unchanged, while surgical time decreased significantly after the first 11 cases, suggesting that the learning curve primarily affects time efficiency rather than accuracy [21]. Collins et al. [22], in a study of 72 patients operated with the NAVIO system, similarly concluded that robotic systems consistently achieved accurate coronal alignment in over 93% of patients, with no apparent learning curve effect on accuracy. These findings reinforce the reliability of robotic assistance in maintaining alignment precision regardless of the surgeon’s prior experience [22]. However, the learning curve may vary across different robotic platforms. Bell et al. [23] analysed 60 NAVIO-assisted procedures and estimated that 29 cases were required to reach a performance plateau. The greatest time reduction occurred during the intraoperative plan review phase, with a mean decrease of 2.1 min [23]. The authors reported full procedural confidence after approximately ten cases [23]. Similarly, Savov et al. [24] evaluated 70 consecutive TKAs: the operative times became comparable to those of conventional TKA after the first 11 robotic cases [24]. Notably, accuracy in joint line obliquity (JLO), joint height, and limb alignment remained unaffected by the learning curve [24]. Likewise, cumulative experience with the robotic system did not influence the accuracy of implant positioning, the posterior condylar offset ratio (PCOR), the posterior tibial slope (PTS), or the restoration of the native joint line, as reported by Kayani et al. [25]. Additional studies confirm the absence of a learning effect on mechanical axis alignment accuracy [25, 26]. Thiengwittayaporn et al. [27] found that the learning curve for NAVIO could be overcome after just seven cases. However, other authors have reported longer learning phases [27]. Vaidya et al. [28], comparing 75 NAVIO-assisted procedures to 25 conventional TKAs, identified a learning curve of approximately 25 cases. In a meta-analysis of 198 studies, Zhang et al. [29] identified five studies focused on the learning curve in robotic TKA [25, 26, 30–32]. All five measured the learning effect in terms of operative time, with case numbers ranging from 7 to 80. Two studies [25, 30] employed Cumulative Sum Control Chart (CUSUM) analysis to identify the inflexion point at which operative efficiency improved. This transition point ranged between 7 and 11 cases, dividing the learning trajectory into an initial acquisition phase and a subsequent proficiency phase. Marchand et al. [26] reported a continuous reduction in mean surgical time during the first year after robotic integration, averaging 19 min. Only 12% of cases exceeded 70 min, indicating improved efficiency compared to conventional TKA by the same surgeons [26]. CUSUM remains a valuable statistical approach to identify precise turning points along learning curves [25, 30, 33]. Finally, Harato et al. [34] investigated 140 TKA procedures, 67 with bicruciate-stabilised and 73 with bicruciate-retaining implants, performed by three experienced surgeons using the NAVIO or CORI systems. They segmented the procedure into eight operative phases and analysed learning curves for each [34]. The steepest learning curve was observed in the bicruciate-retaining group during the bone resection phase (phase 5), with a regression slope of − 0.392, indicating substantial improvement in efficiency [34]. Bicruciate-stabilised procedures showed a similar trend in the same phase (slope − 0.232), albeit to a lesser extent [34]. Bone resection was identified as the most technically demanding and efficiency-sensitive phase across both implant types [34].

All procedures included in the present study were performed by a single high-volume surgeon with extensive prior experience in knee arthroplasty. Before initiating robotic-assisted TKAs, the surgeon completed multiple dedicated training programs, including advanced workshops and cadaveric courses specifically focused on robotic knee surgery. Furthermore, the surgeon has served for many years as the director of a specialised centre for hip and knee arthroplasty, performing approximately 400 total knee arthroplasties annually. This substantial surgical volume and prior structured training likely contributed to minimising the impact of the initial learning curve and ensuring a consistent operative standard from the earliest robotic cases onward. The institution where the surgeries are conducted is accredited by “EndoCert” (EndoCert certificate, Centres of German Endoprosthetic, German Society for Orthopaedics and Traumatology) [35], which supervises and certifies the quality of the surgical procedures.

## Conclusion

The most efficient gains occur early, and operative times stabilise at approximately 90 min after approximately 40 procedures.

## Data Availability

The datasets generated during and/or analysed during the current study are available throughout the manuscript.
